# Public Knowledge and Beliefs Regarding Pharmacy-Based Immunization in Poland—A Nationwide Cross-Sectional Study, 2024

**DOI:** 10.3390/vaccines12080835

**Published:** 2024-07-24

**Authors:** Iwona Wrześniewska-Wal, Justyna Grudziąż-Sękowska, Jarosław Pinkas, Mateusz Jankowski

**Affiliations:** School of Public Health, Centre of Postgraduate Medical Education, 01-826 Warsaw, Poland

**Keywords:** pharmacy-based immunization, trust in pharmacists, influenza, HPV, pharmacy, vaccination, health policy, Poland

## Abstract

Pharmacy vaccinations are a key public health intervention. This study aimed to assess society’s knowledge about pharmacy vaccinations in Poland. A cross-sectional study was conducted from 10 to 13 May 2024 among 1126 adults; the survey questionnaire contained 13 closed questions. Men (OR: 1.32; [1.02–1.70]; *p* < 0.05), and people aged 50–64 (OR: 1.55; [1.05–2.28]; *p* < 0.05), people with higher education (OR:1.74; [1.35–2.26]; *p* < 0.001), and people declaring trust in the pharmacist’s competencies (OR:3.95; [3.03–5.15]; *p* < 0.001) more often declared knowledge of vaccinations in pharmacies. Support for these services was declared by men (OR:1.74; [1.28–2.36]; *p* < 0.001) and people with higher education (OR:1.39; [1.02–1.89]; *p* < 0.05) and participants declaring trust in the pharmacist’s competences (OR:20.30; [14.65–28.11]; *p* < 0.001). Trust in pharmacists was important. People declaring trust in pharmacists were much more willing to get vaccinated against influenza (40.2%) and zoster (38.0%) at a pharmacy and declared that they would vaccinate their children against HPV at a pharmacy (38.8%) compared to people who did not trust the competences of pharmacists (*p* < 0.001). There was a significant difference in the case of influenza. People who trusted pharmacists were five times more likely to declare their willingness to get vaccinated against influenza (*p* < 0.001).

## 1. Introduction

Vaccination is among the most cost-effective and available public health interventions for preventing common infectious diseases [1]. Over the past five decades, this life-saving intervention has prevented approximately 154 million deaths, including approximately 101 million among children [2]. The European Agenda for Vaccination 2030, a document prepared by the WHO Regional Office for Europe, emphasizes the need to seek and consolidate new ways of carrying out vaccinations (including in pharmacies) to ensure a high level of vaccination and the availability of vaccinations (strategic goal) [3]. Searching for new solutions is particularly important due to the need to ensure access to vaccinations for social groups living in poorly connected or economically underdeveloped areas [4].

Currently, there are vaccines against over 20 potentially fatal diseases, and their administration covers both children and adults [2]. Countries are increasingly making vaccinations for children compulsory [1], with recent examples being France, Serbia, Italy, Germany, and Ukraine [5]. While national programs often provide childhood immunization, specific vaccines, dosages, and schedules may vary by geographic region [6]. The European Center for Disease Prevention and Control (ECDC) has a key role in supporting the prevention and control of communicable diseases by promoting best practices in vaccination programming following Regulation (EC) No. 851/2004 [7].

Poland is one of the countries in which compulsory vaccinations of children are supervised by family doctors or pediatricians [8]. Mandatory childhood vaccines are administered in general practitioners’ clinics. Vaccination of adults is voluntary, and the list of recommended vaccines is published annually by public authorities in Poland. Voluntary adult vaccines are administered in general practitioners’ clinics and specialist clinics as well as in pharmacies [8]. Although childhood vaccination rates have declined over the past two decades, they remain relatively high [8]. According to epidemiological reports published by the National Institute of Public Health (NIZP-PZH), uptake of the diphtheria/tetanus/pertussis (DTP) vaccine decreased from 95.6% in 2010 to 85.4% in 2020, and a similar decline was observed with the polio vaccine (95.6% to 85.5%) during the same period [9]. However, for the measles vaccine, regional differences exist, with 61% of Polish provinces falling below the required coverage for herd immunity [10].

However, vaccination of adults in Poland is voluntary and depends on individual choices [11]. This has led to low adult vaccination rates, particularly for influenza [12,13]. To address this gap, Poland has introduced pharmacy-based vaccinations as a strategy to increase adult vaccination rates. This approach has resulted in significantly higher immunization rates in various countries [14], especially for seasonal influenza vaccination in countries such as the United States [15]. In Europe, Ireland implemented pharmacy vaccination in 2002, and countries such as Portugal and Norway have followed suit to varying degrees [11]. Polish pharmacists have expressed great interest in collaborating with physicians to improve patient care through pharmaceutical services [16]. They played a significant role during the pandemic by providing COVID-19 vaccines and influenza vaccines [17].

Pharmacists trained at the Center for Postgraduate Medical Education performed almost 5% of all vaccinations against COVID-19 and 8% of vaccinations against influenza [18]. It is indicated that the introduction of public financing of pharmacy vaccinations is the main reason for the increase in immunization rates in specific age groups [12]. In Poland, we have three vaccinations financed from public funds in pharmacies dedicated to selected groups (influenza, COVID-19, and pneumococci), but the vaccination rate is still low. In the previous flu season (2023/2024), 5% of Polish residents were vaccinated (not only in pharmacies) [19].

This study aimed to assess public knowledge and beliefs regarding pharmacy-based immunization in Poland as well as to identify barriers and opportunities related to the development of pharmacy-based immunization.

## 2. Materials and Methods

### 2.1. Survey Research Design

The survey was conducted from 10 to 13 May 2024. The research tool was the authors’ questionnaire. This study was conducted using the computer-assisted web interview (CAWI) method (study questionnaire available on a dedicated website). This study was approved by the Ethics Committee of the Center for Postgraduate Medical Education in Warsaw (approval number 442/2023 of 13 December 2023).

### 2.2. Study Population

The survey participants were recruited by an opinion polling company [20]. The research company selected respondents from over 100,000 users of the online research platform. The selection of the sample was based on non-probabilistic quota sampling methods, taking into account gender, age, and size of the place of residence, including the regional division of the country. If a selected subject refused to participate in this study, the next one was selected based on a stratification model. The questionnaire was available on a dedicated website and could be accessed only once using a dedicated link. Therefore, the sample of 1126 subjects was representative of the Polish population [21]. This method was previously used in nationwide cross-sectional studies in Poland [22,23]. Nevertheless, over 93% of households in Poland have Internet access and with the stratification model included, i.e., the age range, there is a low risk of bias resulting from the sampling methods and potential low representativeness of older adults.

### 2.3. Measures

The study questionnaire included 13 questions on public knowledge and beliefs regarding pharmacy-based immunization. The study questionnaire (Supplementary Materials) was developed based on a literature review [12,13,16,17].

A pilot study was carried out on a group of 14 adults in Poland, who filled the questionnaire twice within 2 weeks. After the pilot study, 3 questions were revised to simplify the wording in the text.

Awareness of pharmacy-based immunization in Poland: “In your opinion, can you get vaccinated against certain infectious diseases at a pharmacy?” (yes/no).

Support for the possibility of pharmacy-based immunization in Poland: “Do you support the possibility of pharmacy-based immunization in Poland?” with a 5-point Likert scale. Respondents who said definitely yes or rather yes were considered as those who support pharmacy-based immunization.

History of vaccination in pharmacy: “Have you ever had a vaccination at a pharmacy (e.g., against COVID-19 or flu)?” (yes/no).

Trust in pharmacist’s competencies: “Vaccinations in pharmacies are performed by pharmacists who are trained and have appropriate qualifications” with a 5-point Likert scale. Respondents who said definitely yes or rather yes were considered as those who declared trust in the pharmacist’s competencies.

Sociodemographic variables: The financial status of the family was self-reported using three categories: good/moderate/bad. Currently employed or self-employed participants were classified as those with an active occupational status. Unemployed participants, pensioners, and students were classified as those with a passive occupational status.

### 2.4. Statistics

Data analysis was completed using SPSS version 28 (IBM, Armonk, NY, USA). Categorial variables were presented with frequencies and proportions. Cross-tabulations with chi-squared tests were prepared to compare categorical variables. Logistic regression analyses were used to analyze the relationships between sociodemographic variables and dependent variables in different models: (1) awareness of pharmacy-based immunization in Poland; (2) support for the possibility of pharmacy-based immunization in Poland; (3) getting vaccinated at a pharmacy in the past; and (4) willingness to get vaccinated against flu in the pharmacy. In the bivariable analysis, all variables were considered independently. In multivariable models, only variables that were statistically significant in the bivariable analysis were included. The strength of the associations was presented with odds ratio (OR) and 95% confidence interval (95%CI). The statistical significance criterion was based on *p* < 0.05.

## 3. Results

Responses were received from 1126 individuals aged 18–89 years; 53.5% were women. Table 1 presents the participants’ characteristics.

### 3.1. Participants’ Knowledge and Beliefs Regarding Pharmacy-Based Immunization in Poland

Among the respondents (Table 2), over 57.2% knew that vaccinations against infectious diseases can be administered at pharmacies. Similar results were obtained when asked about support for vaccinations in pharmacies (57.6% “definitely yes” or “rather yes”). Respondents indicated that, if there were such an opportunity, they would be willing to get vaccinated against influenza (40.2%) and zoster (38.0%) at a pharmacy. They also declared that they would vaccinate their children against HPV at the pharmacy (38.8%). Moreover, a large part of the respondents indicated that they had confidence in pharmacists (63.8% “definitely yes” and “rather yes”), even though only less than a quarter of the respondents declared that they had been vaccinated at a pharmacy in the past. Respondents indicated the most important advantages of vaccination in a pharmacy: shortening the time spent on vaccinations (45.6%), location of the pharmacy close to home (40.9%), and the possibility of purchasing the vaccine and getting vaccinated in one place (40%). The barriers included fear of complications after vaccination in the pharmacy (34.6%), lack of privacy (e.g., a separate room for vaccination) (33.7%), and pharmacists’ competencies and skills in performing vaccinations (31.8%).

### 3.2. Sociodemographic Differences in Participants’ Knowledge and Beliefs Regarding Pharmacy-Based Immunization

The participants’ knowledge and beliefs regarding pharmacy-based immunization by their sociodemographic factors is presented in Table 3. Men compared to women more often declared positive attitudes toward pharmacy-based immunization (*p* < 0.05). The highest awareness and support for pharmacy-based immunizations was observed among Poles aged 65 and over (*p* < 0.05). People with higher education (64.7%) (*p* < 0.001) and those who had children (59.4%) had greater knowledge about vaccinations in pharmacies than other groups of respondents. Support for vaccination services in pharmacies was more often expressed by men (63.2%) (*p* < 0.001), with higher education (*p* < 0.001), living in cities (60.3%). Respondents who declared that they had been vaccinated at a pharmacy in the past were people with higher education (26.3%) and professionally active (25.3%). Importantly, these were participants who lived in urban areas (26.4%) (*p* < 0.001) (Table 3).

The participants’ attitudes toward willingness to get vaccinated in a pharmacy against selected infectious diseases are presented in Table 4. Men more often declared willingness to get vaccinated in pharmacies against all the examined infectious diseases (influenza—46.9%, COVID-19—43.1%, pneumococci—40.6%, zoster—44.1%), compared to women (*p* < 0.001) (Table 4). Moreover, people aged 65+ more often declared willingness to be vaccinated against influenza (50.2%) (*p* < 0.001) and against COVID-19 (52.6%) (*p* < 0.001). Respondents living in rural areas showed a lower willingness to get vaccinated against all the surveyed diseases compared to people living in urban areas. People living in the urban areas more often declared their willingness to be vaccinated against pneumococci (37.3%) (*p* < 0.003). Study participants who declared confidence in the competences of pharmacists more often declared willingness to get vaccinated against all the diseases tested compared to people who did not trust the competences of pharmacists (*p* < 0.001).

### 3.3. Factors Associated with Participants’ Knowledge and Beliefs Regarding Pharmacy-Based Immunization in Poland

In multivariable logistic regression models, men (OR:1.32; [1.02–1.70]; *p* < 0.05), those aged 50–64 years (OR:1.55; [1.05–2.28]; *p* < 0.05), those having higher education (OR:1.74; [1.35–2.26]; *p* < 0.001), and participants who declared trust in pharmacist’s competencies (OR:3.95; [3.03–5.15]; *p* < 0.001) were more likely to declare awareness of pharmacy-based immunization in Poland (Table 5). Men (OR:1.74; [1.28–2.36]; *p* < 0.001), those having higher education (OR:1.39; [1.02–1.89]; *p* < 0.05), and participants who declared trust in pharmacist’s competencies (OR:20.30; [14.65–28.11]; *p* < 0.001) were more likely to declare support for the possibility of pharmacy-based immunization in Poland (Table 5). Participants aged 35–49 (OR:1.59; [1.04–2.43]; *p* < 0.05), those living in urban areas (OR:1.68; [1.22–2.32]; *p* < 0.01), occupationally active individuals (OR:1.57; [1.06–2.32]; *p* < 0.05), and participants who declared trust in pharmacist’s competencies (OR:3.50; [2.43–5.03]; *p* < 0.001) were more likely to declare getting vaccinated at a pharmacy in the past (Table 5).

Men (OR:1.76; [1.35–2.31]; *p* < 0.001), those aged 65 and over (OR:1.68; [1.09–2.57]; *p* < 0.05), and participants who declared trust in pharmacist’s competencies (OR:9.65; [6.86–13.58]; *p* < 0.001) were more likely to declare willingness to get vaccinated against flu in the pharmacy (Table 6).

## 4. Discussion

A survey of public attitudes and beliefs in Poland toward vaccinations in pharmacies shows that adults were aware (57.2%) that they can be vaccinated against infectious diseases in pharmacies and that they also support these services (57.6%). Respondents indicated that if there were such an opportunity, they would be willing to get vaccinated against influenza (40.2%) and zoster (38.0%) at a pharmacy. They declared that they would vaccinate their children against HPV at the pharmacy (38.8%). Respondents indicated the advantages of vaccination in a pharmacy, such as shortening the time spent on vaccinations (45.6%), location of the pharmacy close to home (40.9%), and the possibility of purchasing the vaccine and getting vaccinated in one place (40%). They considered the barriers to be fear of complications after vaccination in the pharmacy (34.6%), lack of privacy (e.g., a separate room for vaccination) (33.7%), and pharmacists’ competencies (31.8%). The study participants who declared high confidence in the competencies of pharmacists and were much more willing to get vaccinated against all the diseases examined, especially in the case of influenza, compared to people who did not trust the competencies of pharmacists.

Precedents from other countries such as Australia, Canada, and USA [24,25,26,27] and the European countries Great Britain, Wales [28,29], and Portugal [27,30] show that, when pharmacists were involved in the vaccination process, regardless of their role (educator, administrator) [31] or the type of vaccine administered (e.g., influenza, pneumococcal) [32], more people were vaccinated compared to clinic vaccine administration. The introduction of vaccinations in pharmacies and their administration by pharmacists significantly improved the vaccination rate, while reducing the costs of treating infections and the resulting complications, including in the group of seniors—vaccinations against influenza [17,33]—and in the group of teenagers—vaccinations against HPV [34]. In Poland, vaccinations have always been performed in clinics. Only the fight against the pandemic and the introduction of vaccinations against COVID-19 and influenza to Polish pharmacies in 2021 drew the attention of the entire society to the new role and opinions of pharmacists in the field of vaccinations [35].

This study shows that, in Poland, the new role of pharmacists was noticed primarily by men, who had more knowledge that vaccinations can be administered at a pharmacy (61%) than women. This result was related to higher education and living in a large city in which access to a pharmacy is easy. The study result confirms that open pharmacies are well located and prepared to offer health services [30]. Similar results have come from a study in Nova Scotia, in which almost half of the study participants indicated that their experience with vaccinations at a pharmacy was better than at clinics due to convenience and speed [36], similar to the case of flu vaccinations in Estonia [37]. A pharmacy “close to home” [38] and open at convenient hours allows people who do not have time to go to a clinic, work a lot, and finish work late to get vaccinated quickly and conveniently. Research in the USA shows that patients had a much greater chance of being vaccinated at a pharmacy than at a clinic, because pharmacists performed 30.5% of vaccinations in the afternoon (outside the clinic’s opening hours), 17.4% on weekends, 10.2% in the evenings, and 2.9% on holidays [39]. The respondents of the presented study highly assessed the benefits related to reducing the time spent on vaccination at the pharmacy (45.6%) and locating the pharmacy close to home (40.9%). Analogous results are shown by studies from Great Britain, in which patients prefer to be vaccinated at a pharmacy because it is difficult to make an appointment at a clinic, it is further from home, or they simply prefer to go to the pharmacy [40]. An important barrier identified in this study was access to a pharmacy. Respondents who indicated the countryside as their place of residence also indicated the advantages of vaccination in a pharmacy: shortening the time spent on vaccination (41%) and the possibility of vaccination in one place (36%).

Respondents from large cities (24.6%) and professionally active respondents (25.3%) declared that they not only knew about vaccinations in pharmacies but had already used this service in the past. The data containing information on vaccinations in Polish pharmacies come from the P1 platform operated by the e-Health Center, collected since 2020, indicating that over 2.6 million vaccinations against COVID-19 were carried out in Poland during the pandemic [18]. This means that pharmacists working at pharmacy and non-pharmacy vaccination points carried out almost 13% of all vaccinations against COVID-19 and influenza in Poland [18]. However, this opportunity was not properly used. In the 2023/2024 flu season, 5% of Polish residents were vaccinated in pharmacies and clinics [19].

In addition to the barriers described above regarding the organization of vaccinations in pharmacies, financial barriers must be removed. Poland is a country that has reimbursement systems for voluntary vaccinations. Currently, pharmacies can provide protective vaccinations financed by public funds against influenza for people over 65 years of age and pregnant women, against COVID-19 for adults, and against pneumococci for people over 65 years of age in a single-dose system [41]. Moreover, in the case of influenza, the vaccine is reimbursed to all adults aged 18–64 (50% reimbursement).

For other patient groups, the abovementioned vaccinations can be performed in pharmacies for a fee. However, the problem arises in the context of a prescription entitled to reimbursement and a prescription with the right to free vaccinations (prescription with the annotation “S”—senior and “DZ”—child) issued by a family doctor. Only after receiving the prescription can the patient use their rights and go to the pharmacy for vaccination. It seems that this barrier should be eliminated as soon as possible. The presented study clearly shows that the respondents men over the age of 65, as in other countries, e.g., Australia [37] and Wales [42], expressed their willingness to get vaccinated against influenza. Respondents indicated the possibility of vaccination in one place (40%) as an advantage of vaccination in a pharmacy. The optimal situation is a situation in which the patient’s qualifications, prescription for a reimbursed prescription, and vaccination take place in one place, e.g., pharmacy.

Respondents indicated that, if such an opportunity existed, they would be willing to vaccinate against influenza (46.9%), pneumococcal disease (40.6%), and shingles (44.1%) at a pharmacy, and they would be willing to vaccinate their children against HPV (44.8%). However, for the latter two vaccinations, patients cannot be vaccinated in pharmacies. It is worth noting that this study indicates that the acceptability of the zoster and HPV vaccinations is at a level similar to that of influenza and pneumococcal vaccinations. This indicates that it is worth considering the expansion of pharmacy vaccination options to include shingles and HPV vaccinations. The incentive for this type of activity comes from research conducted 10 years ago in the USA. The average direct costs incurred for vaccinating an adult against herpes zoster, pneumococcus, and influenza were lower in pharmacies than in doctor’s offices and other medical facilities [43].

This success is because pharmacists have been vaccinating older adults in pharmacies for almost 20 years and have become familiar with the guidelines [44] and practices for vaccination against influenza, pneumococci, shingles, and tetanus [45]. Our study shows that, in Poland, there is also potential to implement vaccination programs in pharmacies covering care for special groups (e.g., seniors and youth). From 2023, a nationwide vaccination program against HPV has been implemented in Poland. Research shows that the HPV vaccine enjoys good social acceptance, but information on this subject should be addressed to the parents of children eligible for this vaccination, and it requires the involvement of a family doctor [46].

Experience from the USA shows that pharmacies can become an effective place for vaccinating young people against HPV if public awareness of the competencies and training of pharmacists in the field of HPV vaccination increases [47,48] and if there is cooperation between the pharmacist and the doctor [49,50]. It seems that, in the case of HPV vaccination in Poland, as in other countries, pharmacists can play the role of teachers of both young people and parents.

The presented study indicates that the study participants trust pharmacists (80%) because they believe that Polish pharmacists have appropriate qualifications to carry out vaccinations (71% “definitely yes” and “rather yes”). There are also expectations regarding high qualifications of pharmacists in countries where, as in Poland (e.g., in Saudi Arabia), pharmacies are just starting to offer services other than dispensing drugs, such as diabetes education, health promotion and drug therapy management, and vaccinations [51].

This shows that high-income countries are considering expanding pharmacy services to include vaccinations. In contrast, the vast majority of low- and middle-income countries still lag in strengthening pharmaceutical care [52]. Recommendations for these countries include (1) the mandatory presence of pharmacists with higher education in community pharmacies and (2) the training of pharmacists in clinical skills, vaccinations, and programs for minor ailments [52]. The presented study shows another important conclusion: the acceptance of vaccinations in pharmacies and the willingness to use these services increases with increasing trust in pharmacists. The study participants trusted pharmacists. Respondents who showed higher knowledge about vaccinations in pharmacies declared this trust at the level of 69.7% (*p* < 0.001); those who declared support for vaccination services in pharmacies were at the level of 80.5% (*p* < 0.001), and those who in the past had vaccinations at a pharmacy were at 29.5% (*p* < 0.001).

### 4.1. Limitations

This study has limitations related to its cross-sectional design. The research method is based on assisted Internet interviews, so people without Internet access were not included in the study population. Nevertheless, over 93% of households in Poland have Internet access and with the stratification model included, i.e., the age range, there is a low risk of bias resulting from the sampling methods and potential low representativeness of older adults. Despite the pilot and the use of well-known questions related to the awareness and perception of vaccinations in pharmacies and the willingness to get vaccinated at a pharmacy, there was no interaction with respondents, and although minimal, there is a risk of systematic error. Moreover, this study included only four vaccinations recommended for adults, and the question about HPV vaccination concerned the willingness to vaccinate a child. However, the authors found it useful to present these results and thus contribute to the discussion on the impact of pharmacy vaccinations on reducing the burden of disease at the community level across Europe.

### 4.2. Practical Implications

Determining the barriers and advantages affecting the provision of vaccination services in pharmacies in Poland will affect the expansion of developing vaccination services in Polish pharmacies. The planned changes include patients’ acceptance of the introduction of further vaccinations in pharmacies: against zoster and HPV. Moreover, the study results constitute a recommendation for low- and middle-income countries in which vaccination services in pharmacies are just being launched. The key recommendation resulting from this study concerns building vaccination strategies in pharmacies based on trust in pharmacists. The greater patients’ trust in pharmacists, the greater their willingness to vaccinate and obtain these services in pharmacies.

## 5. Conclusions

Pharmacists can play an important role in increasing access to vaccines and improving vaccination coverage. Polish patients are ready to expand the scope of vaccinations against shingles and HPV in pharmacies. Vaccinations in pharmacies have gained acceptance primarily due to the benefits of reducing the time spent on vaccination in the pharmacy and locating the pharmacy close to home. Pharmacists are perceived as highly qualified people, well prepared to carry out vaccinations. This study shows that social trust is crucial. The construction of a vaccination system in pharmacies must be accompanied by a campaign aimed at increasing trust in pharmacists.

## Figures and Tables

**Table 1:** Participants’ characteristics (*n* = 1126).

Variable	*n*	%
Sex		
Women	602	53.5
Men	524	46.5
Age group		
18–34	263	23.4
35–49	323	28.7
50–64	329	29.2
65+	211	18.7
Higher education		
Yes	518	46.0
No	608	54.0
Currently married		
Yes	613	54.4
No	513	45.6
Place of residence		
Rural area	705	62.6
Urban area	421	37.4
Having children		
Yes	766	68.0
No	360	32.0
Occupational activity		
Active	683	60.7
Passive	443	39.3
Economic status of the family		
Good	542	48.1
Moderate	414	36.8
Bad	170	15.1

**Table 2:** Participants’ knowledge and beliefs regarding the pharmacy-based immunization in Poland (*n* = 1126).

Variable	Total (*n* = 1126)	
	*n*	%
In your opinion, can you get vaccinated against certain infectious diseases at a pharmacy?		
Yes	644	57.2
No	482	42.8
Do you support the possibility of pharmacy-based immunization in Poland?		
Definitely yes	253	22.5
Rather yes	395	35.1
Rather no	159	14.1
Definitely no	185	16.4
Difficult to tell	134	11.9
Have you ever had a vaccination at a pharmacy (e.g., against COVID-19 or flu)?		
Yes	254	22.6
No	872	77.4
If it were possible, would you get vaccinated at a pharmacy against…? (only positive answers “yes” presented)		
Flu	453	40.2
COVID-19	425	37.7
Pneumococci	383	34.0
Zoster	428	38.0
Vaccination against HPV for your child	437	38.8
Do you agree with the statement: “Vaccinations in pharmacies are performed by pharmacists who are trained and have appropriate qualifications”? (Trust in pharmacist’s competencies)		
Definitely yes	293	26.0
Rather yes	426	37.8
Rather no	82	7.3
Definitely no	66	5.9
Difficult to tell	259	23.0
In your opinion, what are the advantages of pharmacy-based immunization in Poland? (multiple-choice format; only positive answers “yes” presented)?		
Reducing the time spent on vaccination	514	45.6
Pharmacy location close to home	460	40.9
Possibility to buy vaccine and get vaccinated in one place (without the need to transport the purchased vaccine to a medical clinic)	450	40.0
Possibility to get vaccinated when purchasing medicines (possibility of vaccination when visiting a pharmacy)	355	31.5
Professional pharmacist service	255	22.6
In your opinion, what are the barriers to the widespread implementation of pharmacy-based immunization in Poland? (multiple-choice format; only positive answers “yes” presented)?		
Fear of complications after vaccinations at the pharmacy (inability to obtain professional medical care)	390	34.6
Lack of privacy (e.g., location of the vaccination room)	380	33.7
Competences of pharmacists and skills in performing vaccinations	358	31.8
Lack of knowledge about the possibility of vaccination in a pharmacy	269	23.9
Availability of vaccines in the pharmacy	155	13.8

**Table 3:** Participants’ knowledge and beliefs regarding the pharmacy-based immunization by sociodemographic factors (*n* = 1126).

Variable	Awareness of Pharmacy-Based Immunization in Poland (Positive Responses—Yes)			Support for the Possibility of Pharmacy-Based Immunization in Poland (Positive Responses—Definitely Yes and Rather Yes)			Getting Vaccinated at a Pharmacy in the Past (Positive Responses—Yes)		
	*n*	%	*p*	*n*	%	*p*	*n*	%	*p*
Sex									
Women	326	54.2	**0.03**	317	52.7	**<0.001**	123	20.4	0.07
Men	318	60.7		331	63.2		131	25.0	
Age group									
18–34	130	49.4	**<0.001**	139	52.9	**0.02**	45	17.1	0.07
35–49	173	53.6		173	53.6		85	26.3	
50–64	205	62.3		200	60.8		74	22.5	
65+	136	64.5		136	64.5		50	23.7	
Higher education									
Yes	335	64.7	**<0.001**	325	62.7	**0.001**	136	26.3	**0.01**
No	309	50.8		323	53.1		118	19.4	
Currently married									
Yes	363	59.2	0.1	363	59.2	0.2	142	23.2	0.6
No	281	54.8		285	55.6		112	21.8	
Place of residence									
Rural area	227	53.9	0.09	223	53.0	**0.02**	68	16.2	**<0.001**
Urban area	417	59.1		425	60.3		168	26.4	
Having children									
Yes	455	59.4	**0.03**	440	57.4	0.9	184	24.0	0.09
No	189	52.5		208	57.8		70	19.4	
Occupational activity									
Active	385	56.4	0.5	383	56.1	0.2	173	25.3	**0.01**
Passive	259	58.5		265	59.8		81	18.3	
Economic status of the family									
Good	317	58.5	0.06	332	61.3	0.05	125	23.1	0.9
Moderate	244	58.9		226	54.6		92	22.2	
Bad	83	48.8		90	52.9		37	21.8	
Trust in pharmacist’s competencies									
Yes	501	69.7	**<0.001**	579	80.5	**<0.001**	212	29.5	**<0.001**
No	143	35.1		69	17.0		42	10.3	

Statistically significant values are bolded.

**Table 4:** Participants’ attitudes toward willingness to get vaccinated in a pharmacy against selected infectious diseases (*n* = 1126).

Variable	If It Were Possible, Would You Get Vaccinated at a Pharmacy Against…? (Only Positive Answers “Yes” Presented)														
	Flu			COVID-19			Pneumococci			Zoster			Vaccination against HPV for Your Child		
	*n*	%	*p*	*n*	%	*p*	*n*	%	*p*	*n*	%	*p*	*n*	%	*p*
Sex															
Women	207	34.4	**<0.001**	199	33.1	**<0.001**	170	28.2	**<0.001**	197	32.7	**<0.001**	202	33.6	**<0.001**
Men	246	46.9		226	43.1		213	40.6		231	44.1		235	44.8	
Age group															
18–34	84	31.9	**<0.001**	68	25.9	**<0.001**	82	31.2	0.1	91	34.6	0.2	103	39.2	0.9
35–49	122	37.8		112	34.7		101	31.3		114	35.3		123	38.1	
50–64	141	42.9		134	40.7		114	34.7		135	41.0		132	40.1	
65+	106	50.2		111	52.6		86	40.8		88	41.7		79	37.4	
Higher education															
Yes	209	40.3	0.9	214	41.3	**0.02**	186	35.9	**0.2**	197	38.0	0.9	208	40.2	0.4
No	244	40.1		211	34.7		197	32.4		231	38.0		229	37.7	
Currently married															
Yes	265	43.2	**0.03**	246	40.1	0.07	211	34.4	0.8	231	37.7	0.8	231	37.7	0.4
No	188	36.6		179	34.9		172	33.5		197	38.4		206	40.2	
Place of residence															
Rural area	157	37.3	0.1	139	33.0	**0.01**	120	28.5	**0.003**	141	33.5	**0.02**	143	34.0	**0.01**
Urban area	296	42.0		286	40.6		263	37.3		287	40.7		294	41.7	
Having children															
Yes	318	41.5	0.2	303	39.6	0.07	258	33.7	0.7	285	37.2	0.4	293	38.3	0.6
No	135	37.5		122	33.9		125	34.7		143	39.7		144	40.0	
Occupational activity															
Active	262	38.4	0.1	238	34.8	**0.01**	225	32.9	0.3	255	37.3	0.6	269	39.4	0.6
Passive	191	43.1		187	42.2		158	35.7		173	39.1		168	37.9	
Economic status of the family															
Good	223	41.1	0.6	218	40.2	0.2	190	35.1	0.7	206	38.0	0.9	210	38.7	0.9
Moderate	167	40.3		149	36.0		139	33.6		160	38.6		161	38.9	
Bad	63	37.1		58	34.1		54	31.8		62	36.5		66	38.8	
Trust in pharmacist’s competencies															
Yes	406	56.5	**<0.001**	381	53.0	**<0.001**	347	48.3	**<0.001**	378	52.6	**<0.001**	379	52.7	**<0.001**
No	47	11.5		44	10.8		36	8.8		50	12.3		58	14.3	

Statistically significant values are bolded.

**Table 5:** Factors associated with participants’ knowledge and beliefs regarding the pharmacy-based immunization in Poland (*n* = 1126).

	Factors Associated with Participants’ Knowledge and Beliefs Regarding the Pharmacy-Based Immunization in Poland					
Variable	Awareness of Pharmacy-Based Immunization in Poland		Support for the Possibility of Pharmacy-Based Immunization in Poland		Getting Vaccinated at a Pharmacy in the Past	
	Bivariable Logistic Regression	Multivariable Logistic Regression	Bivariable Logistic Regression	Multivariable Logistic Regression	Bivariable Logistic Regression	Multivariable Logistic Regression
	OR (95%CI)	OR (95%CI)	OR (95%CI)	OR (95%CI)	OR (95%CI)	OR (95%CI)
Sex						
Women	Reference	Reference	Reference	Reference	Reference	
Men	1.31 (1.04–1.66) *	1.32 (1.02–1.70) *	1.54 (1.21–1.96) ***	1.74 (1.28–2.36) ***	1.30 (0.98–1.72)	
Age group						
18–34	Reference	Reference	Reference	Reference	Reference	Reference
35–49	1.18 (0.85–1.64)	1.07 (0.74–1.53)	1.03 (0.74–1.43)	0.81 (0.53–1.24)	1.73 (1.15–2.60) **	1.59 (1.04–2.43) *
50–64	1.69 (1.22–2.35) **	1.55 (1.05–2.28) *	1.38 (0.99–1.92)	1.06 (0.69–1.62)	1.41 (0.93–2.12)	1.35 (0.88–2.07)
65+	1.86 (1.28–2.69) **	1.47 (0.95–2.27)	1.62 (1.12–2.35) *	1.02 (0.63–1.63)	1.50 (0.96–2.36)	1.66 (0.97–2.84)
Higher education						
Yes	1.77 (1.39–2.25) ***	1.74 (1.35–2.26) ***	1.49 (1.17–1.89) **	1.39 (1.02–1.89) *	1.48 (1.12–1.96) **	1.24 (0.93–1.67)
No	Reference	Reference	Reference	Reference	Reference	Reference
Currently married						
Yes	1.20 (0.95–1.52)		1.16 (0.92–1.47)		1.08 (0.82–1.43)	
No	Reference		Reference		Reference	
Place of residence						
Rural area	Reference		Reference	Reference	Reference	Reference
Urban area	1.24 (0.97–1.58)		1.35 (1.06–1.72) *	1.25 (0.91–1.71)	1.86 (1.37–2.53) ***	1.68 (1.22–2.32) **
Having children						
Yes	1.32 (1.03–1.70) *	1.04 (0.77–1.41)	0.99 (0.77–1.27)		1.31 (0.96–1.78)	
No	Reference	Reference	Reference		Reference	
Occupational activity						
Active	0.92 (0.72–1.17)		0.86 (0.67–1.09)		1.52 (1.13–2.04) **	1.57 (1.06–2.32) *
Passive	Reference		Reference		Reference	Reference
Economic status of the family						
Good	1.48 (1.05–2.09) *	1.25 (0.85–1.82)	1.41 (0.99–1.99)		1.08 (0.71–1.63)	
Moderate	1.50 (1.05–2.15) *	1.38 (0.94–2.04)	1.07 (0.75–1.53)		1.03 (0.67–1.58)	
Bad	Reference	Reference	Reference		Reference	
Trust in pharmacist’s competencies						
Yes	4.24 (3.28–5.49) ***	3.95 (3.03–5.15) ***	20.26 (14.7–27.8) ***	20.30 (14.65–28.11) ***	3.63 (2.54–5.20) ***	3.50 (2.43–5.03) ***
No	Reference	Reference	Reference	Reference	Reference	Reference

* *p* < 0.05; ** *p* < 0.01; *** *p* < 0.001.

**Table 6:** Factors associated with willingness to vaccinate against flu in pharmacy among adults in Poland (*n* = 1126).

Variable	Willingness to Vaccinate against Flu in the Pharmacy	
	Bivariable Logistic Regression	Multivariable Logistic Regression
	OR (95%CI)	OR (95%CI)
Sex		
Women	Reference	Reference
Men	1.69 (1.33–2.15) ***	1.76 (1.35–2.31) ***
Age group		
18–34	Reference	Reference
35–49	1.29 (0.92–1.82)	1.16 (0.78–1.71)
50–64	1.60 (1.14–2.24) **	1.33 (0.90–1.96)
65+	2.15 (1.48–3.13) **	1.68 (1.09–2.57) *
Higher education		
Yes	1.01 (0.80–1.28)	
No	Reference	
Currently married		
Yes	1.32 (1.04–1.67) *	1.08 (0.82–1.43)
No	Reference	Reference
Place of residence		
Rural area	Reference	
Urban area	1.22 (0.95–1.56)	
Having children		
Yes	1.18 (0.92–1.53)	
No	Reference	
Occupational activity		
Active	0.82 (0.64–1.05)	
Passive	Reference	
Economic status of the family		
Good	1.19 (0.83–1.69)	
Moderate	1.15 (0.80–1.66)	
Bad	Reference	
Trust in pharmacist’s competencies		
Yes	9.94 (7.09–13.93) ***	9.65 (6.86–13.58) ***
No	Reference	Reference

* *p* < 0.05; ** *p* < 0.01; *** *p* < 0.001.

## Data Availability

Data are available from the corresponding author on reasonable request.

## Supplementary Materials

The following supporting information can be downloaded at https://www.mdpi.com/article/10.3390/vaccines12080835/s1, Study questionnaire.
