# Supplementary material for: Public Knowledge and Beliefs Regarding Pharmacy-Based Immunization in Poland—A Nationwide Cross-Sectional Study, 2024
Source: Vaccines (Basel). 2024 Jul 24;12(8):835. doi: 10.3390/vaccines12080835 (PMC11359024; doi:10.3390/vaccines12080835)
Supplement: Supplementary file 1 [file vaccines-12-00835-s001.zip › vaccines-3092077-supplementary.pdf]

STUDY QUESTIONNAIRE

Read the description below, then answer the questions.

[Q1] In your opinion, can you get vaccinated against certain infectious diseases at a pharmacy?

\hv

Q r

[Q2] Do you support the possibility of pharmacy based immunization in Poland?

definitely yes

rather yes

rather no

definitely no

difficult to tell

[Q3] Have you ever had a vaccination at a pharmacy (e.g. against COVID-19 or flu)?

Yes

No

[Q4] If it were possible, would you get vaccinated at a pharmacy against ?
[only positive answers "yes" presented]

a. I will get vaccinated against flu	\HV	Q R
b. I will get vaccinated against COVID 19	\HV	Q R
c. I will get vaccinated against pneumococci	\HV	Q R
d. I will get vaccinated against shingles	\HV	Q R
e. I will vaccinate my child against HPV	\HV	Q R

[Q5] Do you agree with the statement: Vaccinations in pharmacies are performed by pharmacists who are trained and have appropriate qualifications [Trust in pharmacist's competencies]

definitely yes

rather yes

rather no

definitely no
difficult to tell

[Q6] In your opinion, what are the advantages of pharmacy based immunization in Poland? [multiple-choice format; positive answers]:

a. possibility to buy vaccine and get vaccinated in one place (without the need to transport the purchased vaccine to a medical clinic)
b. reducing the time spent on vaccination
c. possibility to get vaccinated when purchasing medicines (possibility of vaccination when visiting a pharmacy)
d. pharmacy location close to home
e. professional pharmacist service.

[Q7] In your opinion, what are the barriers to the widespread implementation of pharmacy based immunization in Poland? [multiple-choice format; positive answers]:

a. fear of complications after vaccinations at the pharmacy (inability to obtain professional medical care)
b. lack of privacy (e.g., location of the vaccination room)
c. competences of pharmacists and skills in performing vaccinations
d. availability of vaccines in the pharmacy
e. lack of knowledge about the possibility of vaccination in a pharmacy.

Metrics

1. **What is your gender?**
   woman
   man
2. **What age are you?**
   18-24 years old
   25-34 years old
   35-44 years old
   45-54 years old
   55 years or older

   Please indicate your year of age_________
3. **Where do you live? Select the size of the town where you live**
   rural area
   small town (up to 20,000 inhabitants)
   medium-sized city (from 20 to 99 thousand inhabitants)
   big city (from 100 to 500 thousand inhabitants)
   big city (over 500,000 inhabitants)
4. **What is your current education (last school)?**
   primary or secondary school

essential
medium
post-secondary or post-secondary
bachelor degree
completed higher education

5. **How do you generally self-assess the current financial situation of your family?**
bad
hard to say, some bad and some good (moderate)
good

6. **What is your current professional status?**
I work on a contract of employment
I work on civil contract
I am working on a business contract
I run my own business
unemployed
retiree or pensioner
pupil or student
I run the house
other (enter which)

7. **What is your marital status?**
free (single)
married in an informal relationship
other (enter which)

8. **Do you have children?**
Yes
No
(Please indicate how many __)

9. **Do children under 18 live with you?**
Yes
No
(Please indicate how many __)
